# Pregnancy experiences of transgender and gender‐expansive individuals: A systematic scoping review from a critical midwifery perspective

**DOI:** 10.1111/birt.12834

**Published:** 2024-05-20

**Authors:** Elias G. Thomas, Bahareh Goodarzi, Hannah Frese, Linda J. Schoonmade, Maaike E. Muntinga

**Affiliations:** ^1^ Department of Internal Medicine Amsterdam UMC Amsterdam the Netherlands; ^2^ Amsterdam Public Health Amsterdam The Netherlands; ^3^ Department of Ethics, Law and Humanities Amsterdam UMC Amsterdam The Netherlands; ^4^ Midwifery Academy Amsterdam Groningen InHolland Amsterdam The Netherlands; ^5^ Amsterdam UMC Location Vrije Universiteit Amsterdam Midwifery Science Amsterdam the Netherlands; ^6^ Department of General Practice & Elderly Care Medicine, University Medical Center Groningen University of Groningen Groningen the Netherlands; ^7^ Independent Researcher; ^8^ Medical Library Vrije Universiteit Amsterdam Amsterdam the Netherlands

**Keywords:** critical midwifery studies, gender expansive, LGBTIQ+ people, pregnancy, prenatal care, scoping review, transgender

## Abstract

**Background:**

Evidence suggests that transgender and gender‐expansive people are more likely to have suboptimal pregnancy outcomes compared with cisgender people. The aim of this study was to gain a deeper understanding of the role of midwifery in these inequities by analyzing the pregnancy experiences of transgender and gender‐expansive people from a critical midwifery perspective.

**Methods:**

We conducted a systematic scoping review. We included 15 papers published since 2010 that reported on pregnancy experiences of people who had experienced gestational pregnancy at least once, and were transgender, nonbinary, or had other gender‐expansive identities.

**Results:**

Three themes emerged from our analysis: “Navigating identity during pregnancy,” “Experiences with mental health and wellbeing,” and “Encounters in the maternal and newborn care system.” Although across studies respondents reported positive experiences, both within healthcare and social settings, access to gender‐affirmative (midwifery) care and daily social realities were often shaped by trans‐negativity and transphobia.

**Discussion:**

To improve care outcomes of transgender and gender‐expansive people, it is necessary to counter anti‐trans ideologies by “fixing the knowledge” of midwifery curricula. This requires challenging dominant cultural norms and images around pregnancy, reconsidering the way in which the relationship among “sex,” “gender,” and “pregnancy” is understood and given meaning to in midwifery, and applying an intersectional lens to investigate the relationship between gender inequality and reproductive inequity of people with multiple, intersecting marginalized identities who may experience the accumulated impacts of racism, ageism, and classism. Future research should identify pedagogical frameworks that are suitable for guiding implementation efforts.

## 1 INTRODUCTION

Evidence suggests that transgender and gender‐expansive (TGGE) people* are more likely to experience suboptimal reproductive healthcare and worse outcomes compared with cisgender women. Although studies aiming to explain these inequities have identified factors at the individual level, such as dysphoria and social isolation, many findings point to causes within healthcare settings, such as experiences of discrimination, exclusion, and stigma in patient–practitioner interactions, or erasure of transgender and nonbinary identities in the healthcare system.^1^, ^2^, ^3^, ^4^, ^5^, ^6^ Historically, pregnancy and birth have been perceived and narrated as exclusively experienced by cisgender women. Perhaps as a result, providing gender‐affirming reproductive care has been considered complex, and has even been called a “clinical challenge” for maternal and newborn care (MNC) practitioners.^7^ Clinical guidelines and frameworks have been developed to address these issues, and MNC practitioners have been reflecting on what it means to provide responsive reproductive care to TGGE individuals.^5^, ^8^, ^9^, ^10^, ^11^

## 2 THE NEED FOR A CRITICAL MIDWIFERY LENS

Globally, midwives provide a large share of reproductive care.^12^, ^13^ They are in a particularly strong position to provide culturally responsive care to TGGE individuals because they work both inside and outside hospitals and take personal perspectives and circumstances of clients and their loved ones into account.^14^, ^15^ However, recent publications about gendered language reflect controversies and anxieties in the midwifery community about the use of gender‐inclusive language and imagery, and there are debates about who has the right to assign a gender label to a pregnant body.^16^, ^17^, ^18^, ^19^, ^20^ Hetero‐ and cisnormative ideologies in MNC may hinder the widespread adoption and implementation of gender‐inclusive reproductive care.^21^, ^22^ Analyzing the contribution of midwifery in TGGE people's reproductive health inequities, therefore, calls for the use of critical analytical perspectives such as critical midwifery (CM).^23^ Rooted in reproductive justice theory, CM provides a theoretical, methodological, and moral framework to analyze systemic and institutional equity issues in reproductive care from a critical and intersectional perspective.^23^, ^24^ CM also has the potential to offer a strong and academically rigorous counter‐narrative to trans‐exclusive and trans‐negative viewpoints in MNC. Its application requires a reflection on the role of midwifery in shaping, causing, maintaining, sustaining, and (re)producing injustice in the content and conduct of midwifery care.^23^

### 2.1 Aim of this study: transgender and gender‐expansive pregnancy experiences from a critical midwifery perspective

Although several reviews have been published on the reproductive care experiences of TGGE people, these reviews focused on fertility, birth, and lactation care and did not use critical theories as an analytical lens to contextualize and add meaning to their findings.^25^, ^26^, ^27^, ^28^, ^29^, ^30^, ^31^, ^32^, ^33^, ^34^ The use of critical approaches, such as social and health justice frameworks, intersectional analyses, positionality statements by authors, and reflections on institutional aspects of accessibility, is essential to increase insight into the root causes of reproductive health inequities. The aim of this review was, therefore, to analyze the synthesized literature on TGGE experiences from a CM perspective, identify knowledge gaps, and make recommendations for future midwifery research, practice, and education. A key principle of CM is to platform the voices of those whose reproductive health is concerned (epistemic justice).^35^ In this review, we center the lived experiences of TGGE people for several reasons. Not only is prioritizing experiential knowledge imperative for practitioners to adequately consider gender diversity in their everyday work but research into the health of TGGE people has also often ignored TGGE perspectives, and decisions that directly affect TGGE people's bodies and lives are often made without them,^36^ which (re)produces epistemic injustice and perpetuates health inequities.^37^

## 3 METHODS

### 3.1 Study design

We conducted a scoping review using a systematic design for the search and data selection, and inductive thematic analysis for the data analysis and synthesis.^38^

### 3.2 Search strategy and data selection

The literature search was conducted in accordance with the preferred reporting items for systematic reviews and meta‐analysis (PRISMA) statement extension for scoping reviews.^39^, ^40^ A systematic search was performed in the bibliographic databases PubMed, Embase.com, Cinahl (using Ebsco), and Scopus, from inception to May 31, 2023, in collaboration with a medical librarian (LS). Search terms included controlled terms (MeSH in PubMed, Emtree in Embase, and Cinahl Headings) as well as free‐text terms. The following terms were used, including synonyms and closely related words, as index terms or free‐text words: “transgender men,” “prenatal care,” and “experience.” The search was performed without date or language restrictions. The PRISMA statement and full search strategies for all databases can be found in Data S1 and S2.

### 3.3 Data extraction and data analysis

We used a systematic two‐stage screening process to assess the relevance of the papers identified in the search.^41^ In the first stage, three reviewers (ET, HF, and MM) independently screened all potentially relevant titles and abstracts for eligibility using Rayyan software.^42^ Differences in judgment were resolved through a consensus procedure.

We accepted publications published from January 2010 onward that were available in full‐text English, German, and/or Dutch. Studies were included if they met the following criteria: (1) original peer‐reviewed, empirical journal articles, reporting on qualitative, quantitative, or mixed‐methods investigations; (2) reporting on the pregnancy experiences; (3) of TGGE people assigned female at birth, using any transmasculine identity. We excluded studies if it: (1) did not center TGGE individuals' own lived experiences and narratives; and (2) solely addressed the experiences and needs of TGGE people before (e.g., fertility preservation or ART and child wishes) or after pregnancy (e.g., lactation care).

We adopted an inductive thematic synthesis approach to extract and synthesize the data.^43^, ^44^ Data were extracted by the authors (ET, HF, and MM) and discussed and agreed among them (Table 1).

**TABLE 1:** Study characteristics.

Author	Year	Country	Study design	Study population	Main results
Ellis, Simon A. et al.	2015	United States	Online interviews with a self‐administered demographic survey	*N* = 8 Participants identifying as male or gender variant, who carried a pregnancy to term	Participants describe struggles between the internal experience of self (male) and the socio‐normative ideas about pregnancy (female). Heightened connection with their bodies, as well as disconnection, is described by participants. Overall, many participants experienced pregnancy as a lonely process. Participants describe little experience among their caregivers in terms of perinatal care for transgender individuals but were mostly positively surprised by the interaction with healthcare practitioners. Active and passive nondisclosure was used as a coping style during the whole experience of preconception, pregnancy, and birth.
Light, Alexis D. et al.	2014	United States	Cross‐sectional online survey	*N* = 41 Transgender men (self‐identified, assigned female at birth with masculine, transmasculine, transmale, or female‐to‐male identity), age 18 years or older	Participants describe positive as well as negative interactions with the healthcare system. Positive experiences include use of the right pronouns, terminology preferred by pregnant person (e.g., dad), and body parts referred to in the same way as the pregnant person in question. Negative experiences include improper pronoun use, rude treatment, and being turned away. Some participants describe improvements in dysphoria, mostly due to a form of their bodies (finally feeling comfortable). Others experienced increases in dysphoria during pregnancy. Loneliness during pregnancy period is a recurring theme. Participants describe low levels of healthcare practitioner knowledge and a desire for information resources.
Hoffkling, A. et al.	2017	United States	In‐depth semi‐structured interviews	*N* = 10 Transgender men (self‐identified) who had given birth while identifying as male, within last 10 years^a^	Participants describe diverse experiences and values: Empowerment in healthcare, desire for external affirmation of gender and/or pregnancy, access to social support, and degree of outness as male, transgender, and pregnant. Structural barriers that disempower participants, and aspects of care that are safe and empowering. Patient strategies and practitioner behavior affect the level of empowerment and security experienced.
Charter, R. et al.	2018	Australia	Mixed‐methods design: online surveys and semi‐structured interviews	*N* = 25 (survey respondents) *N* = 16 (interview respondents), age 24–46 years old	Pregnancy was viewed by some participants as a functional sacrifice. Withdrawing from testosterone, the growing fecund body, and changes to the chest led to dysphoria. Participants describe exclusion, isolation, and loneliness as part of their experience during pregnancy. They encountered exclusion when interacting with healthcare practitioners.
MacDonald, T. et al.	2016	North America, Australia, and Europe	Semi‐structured online interviews	*N* = 22 Transmasculine individuals (self‐identified) who experienced or were experiencing pregnancy, birth, and infant feeding, aged 24–50 years	Participants with chest masculinization surgery experienced regrowth of chest tissue and other physical changes caused by experiences of body or socially induced dysphoria. The presence of chest tissue was not always cause for dysphoria: functional benefits: healthy baby, attachment, and bonding. Chest swelling is more likely to evoke misgendering or not passing as male than belly growth.
Kirczenow MacDonald, T. et al.	2020	North America, Australia, and Europe	Semi‐structured interviews	*N* = 22 Individuals self‐identifying as transmasculine, gender‐queer, and/or nonbinary and having experienced or experiencing pregnancy	Participants experienced bodily changes beyond their expectations (e.g., bigger belly), including loss of facial hair and regrowth of chest tissue. Pregnancy‐related body changes did not always trigger an increase in dysphoria; some participants viewed pregnancy as a nongendered or masculine experience. “Administrative” difficulties (e.g., insurance coverage of pregnancy for males) also posed an additional stressor. Oppression and discrimination due to other social factors (age, religion, immigration status, etc.) were sometimes also present and hard to untangle from discrimination due to transgender identity.
Bower‐Brown, S. et al.	2020	United Kingdom	Semi‐structured interviews, face‐to‐face, or by phone	*N* = 11 Nonbinary, transmale, or transfemale identities; birth parents, nonbirth parents, or adoptive parents	Participants viewed their bodies as scientific objects during pregnancy, detaching their pregnant body from gender norms or assumptions. Having the experience of pregnancy complicated the disclosure of their gender identity to the outside world for some participants. Nondisclosure led to feelings of frustration for some. Some participants with “intersectional understanding” felt pressure to educate their environment in order to create a better place for their children.
Fischer, O. J.	2020	Canada	Unstructured interviews	*N* = 5 Individuals not identifying as cisgender, having conceived, carried, or birthed a child, mean age of 35	Nonbinary participants experienced more frequent misgendering during pregnancy, challenging their self‐advocacy. Participants experienced pregnancy as gendered, for example, when being approached as “mom” and when buying maternity clothes was challenging. They dealt with it by seeking a gender‐affirming midwife, or by presenting as female to “fit in” and avoid questions. Participants may feel like they were an opportunity for healthcare practitioners to educate themselves, alienating them from their care practitioners, or feeling othered. Because of changes in their care team (other shifts), participants describe having to clarify their gender identity multiple times, creating alienation.
Charlton, B. M. et al.	2020	United States	In‐depth, semi‐structured interviews	*N* = 10 Transmasculine individuals, who experienced a teen or unintended pregnancy, ages 20–59 years	Pregnant participants experienced either misgendering or discrimination because they were read as fat and were stereotyped as such (e.g., being lazy). Sociocultural normativity as a pregnant individual (such as being married) made the pregnancy more “accepted” by family and caregivers.
Falck, F. et al.	2020	Sweden	In‐depth semi‐structured interviews or face to face	*N* = 12 Transmasculine individuals who attended prenatal care and delivered a child	Respondents perceived their practitioners to understand masculinity and pregnancy as incompatible, which resulted in trans‐negative attitudes and experiences of inconsistent care. Respondents felt they had to take charge of their care to ensure their needs during pregnancy were met.
Miller Pyke, C. et al.	2021	United States	Case report with in‐depth interview	*N* = 1 Nonbinary person	Being pregnant during the COVID‐19 pandemic enabled the participant to avoid reactions from society. They experienced the birth center as more respectful compared with a hospital setting, not disclosing their gender identity in hospital.
Asklöv, K. et al.	2021	Sweden	Semi‐structured online interviews	*N* = 9 Transmasculine individuals between 25 and 43 years old	Normalization and confirmation of gender identity lowered barriers to care seeking and enhanced care experiences, as well as gender‐inclusive attitudes and skills (e.g., respectful language use and efficiency) in situations when respondents felt particularly exposed, such as during pelvic examinations.
van Amesfoort, J.E. et al.	2023	Netherlands	Semi‐structured interviews	*N* = 5 Transmasculine individuals between 23 and 35 years old who had given birth	Respondents experienced dysphoria due to physical changes which were the result of pregnancy or cessation of testosterone treatment, and experienced misgendering more often. Some participants described coping with dysphoria by disconnecting and ignoring being pregnant. Gynecological examinations were dreaded by participants, even though staff showed understanding and tried to limit these examinations. Respondents discussed different modes of delivery (including cesarean section) with their healthcare practitioners. Respondents reported that not all healthcare practitioners were willing to provide perinatal care for them as transgender individuals. Respondents also reported positive experiences with healthcare practitioners, such as instructions from their gynecologists to the team about pronouns and scientific research about transmasculine pregnant people. In general, respondents identified the experience of pregnancy and birth as female oriented.
Copeland, M. et al.	2023	Australia	Semi‐structured interviews	*N* = 2	Respondents reported worries before pregnancy about feeling feminized. Interaction with a midwife identifying as queer was reported as a positive experience.
Alvarez, C.F. et al.	2023	United States	Analysis of online posts on a Reddit page for transmasculine parents	N/A	Respondents described the experience of being held to feminine beauty standards when pregnant. Respondents reported difficulty in finding suitable clothes when pregnant, as many maternity clothes were considered feminine. They also reported that apps to track pregnancy and other aiding tools were female oriented. Some respondents reported increased dysphoria and others reported that experiencing their body as functional in terms of pregnancy and labor was experienced as a relief from dysphoria.

^a^ Subset of participants of study by Light et al. (2014), age 18 years or older.

### 3.4 Positionality statement

When applying CM, consideration of the authors’ positionality is essential to contextualize our research.^45^, ^46^ We are a group of individuals of different genders and sexualities, including a transgender man (ET) and a queer‐identified person who uses she/they pronouns (MM). HF is White and German, BG is Dutch‐Iranian, and LJ, ET, and MM are White and Dutch. Although none of the researchers has experience being pregnant as a trans‐masculine person, one of the researchers does have experience with fertility care as a queer person, providing insight into some of the structural barriers within pregnancy‐related care. Furthermore, ET has the lived experience of accessing care as a trans‐masculine person, and the feelings of invisibility, structural cis‐sexism, which may be present when one is dependent on care within a health‐care system largely designed for and executed by cisgender people. At the time of writing, ET is a medical doctor and PhD student, BG is a midwife, educator and post‐doctoral researcher focusing on reproductive justice, HF is a psychologist and gender studies graduate, LS is an information specialist, and MM works as an assistant professor and is obtaining their degree in medicine. These diverse professional backgrounds enable us as a team to apply different lenses to the experience of the respondents in health care. However, as researchers, we bring our personal as well as our professional background into play, not shedding one or the other when reflecting on our findings. As a doctor and a midwife (the shared first authors), our interpretation of the findings as healthcare professionals as well as persons who have personally experienced discrimination is interwoven. Therefore, our thinking is likely impacted by the rules and constraints of the healthcare system as we know them from our daily life and work. Lastly, all of the authors are highly educated and live in a high‐income country. Therefore, we may have less insight into specific challenges at the intersection of TGGE identities and little (access to) formal education, and interpret the findings from the perspective of care in the setting of a high‐income country.

## 4 RESULTS

**FIGURE 1:**
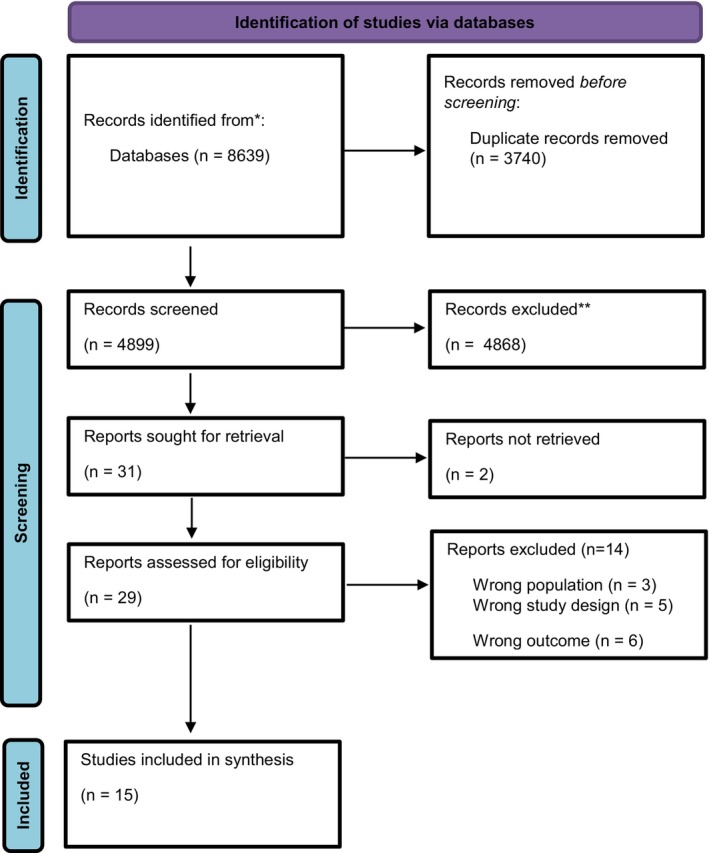
Flowchart of the search and selection procedure of studies. [Colour figure can be viewed at wileyonlinelibrary.com]

The literature search generated 4899 references, of which 15 papers were included for analysis. The flow chart of the search and selection process is presented in Figure 1.

All studies were conducted in the Global North, and four included respondents in Australia (Table 1). There was heterogeneity in gender identities, as well as the extent to which people had experienced medical transition. Most respondents were white. Three interrelated themes emerged from the synthesis: (1) disclosing and navigating identities during pregnancy; (2) experiences with mental health and well‐being; and (3) encounters in the maternal and newborn care system.

### 4.1 Disclosing and navigating identities during pregnancy

Identity‐related experiences of TGGE people during pregnancy varied across studies. Many TGGE individuals who experienced pregnancy mentioned how their changing physique required them to negotiate their transmale or nonbinary identity with their pregnant identity as viewed by themselves and others; this process was sometimes described as painful.^47^, ^48^, ^49^, ^50^, ^51^, ^52^, ^53^, ^54^, ^55^ Redefining masculinity, others viewed their pregnancy as a nongendered or even masculine experience. They established new and gender‐variant pregnancy and parental roles or detached their pregnant body from the notion of gender altogether.^47^, ^48^, ^50^, ^51^

Most TGGE people developed strategies to deal with the way in which other peoples' perceptions of their identity changed when their pregnancy started to show. TGGE individuals reported having to choose between disclosing their pregnancy and/or their gender identity or keeping it hidden.^47^, ^48^, ^49^, ^51^, ^53^, ^56^, ^57^ The degree of comfort in sharing those identities seemed dependent on the degree of safety perceived by an individual within their specific environment.^49^ Transphobia and threats of violence were reported as continuously present, affecting TGGE individuals' sense of safety and agency. Some TGGE people experienced discrimination based on other aspects of their identity, for example, age, religion, and immigration status.^47^, ^51^

Ellis and colleagues (2015) identified three strategic approaches to disclosure: active nondisclosure, passive nondisclosure, and self‐disclosure. Individuals who self‐disclose choose to reveal their pregnancy fully. Some TGGE people disclosed their pregnancy to find social support and affirmation in their daily environment, while others did so to have their gender identity and pregnancy affirmed within the MNC system.^49^, ^50^, ^58^, ^59^ Many TGGE people had no choice other than disclosure: people with a history of gender‐affirming hormone therapy are often obliged to disclose their natal sex, increasing vulnerability to transphobic abuse within the healthcare system, or access to pregnancy care.^51^, ^57^ Some TGGE people, therefore, chose to conceal their pregnancy entirely or to reveal their pregnancy to loved ones and medical practitioners only.^52^ Nondisclosure involved, for instance, intentionally presenting as a cisgender woman when going out in public.^50^, ^59^ While this strategy enhanced feelings of safety, it also decreased affirmation of identity, and as a result, in some individuals, worsened dysphoria and increased feelings of isolation and loneliness.^48^, ^58^ Some respondents chose to “go stealth,”† presenting to incline others to read them as a cisgender male, for instance, by concealing their pregnant stomach and chest, or presenting as a “fat man.”^47^, ^48^, ^51^, ^57^, ^59^ For some TGGE people, going stealth meant less external affirmation of the pregnancy, which limited their access to social and emotional support or physical assistance.^59^ For TGGE individuals who did not want to be read as cisgender female but could not or did not want to go stealth, active nondisclosure provoked—sometimes welcome—feelings of isolation, and some did not leave their house much during their pregnancy.^52^, ^54^, ^59^ Respondents who chose to passively nondisclose did not actively alter or conceal their appearance but left others to assume their gender identity.^49^

### 4.2 Experiences with mental health and well‐being

Two health‐related themes emerged from the literature analysis: experiences with gender dysphoria during pregnancy, and feelings of isolation and loneliness. The health experiences of TGGE people during their pregnancy varied depending on personal characteristics such as disclosure preferences, and structural circumstances such as trans‐negative environments in healthcare and elsewhere.^60^ The pursuit of gestational pregnancy requires TGGE individuals to pause their testosterone intake. This pause was reported by authors as challenging to their respondents' mental health, particularly in terms of flare‐ups of dysphoric feelings.^47^, ^48^, ^51^, ^52^, ^56^, ^57^, ^59^ Physical changes during pregnancy, including regrowth of chest tissue, widening of hips, and reduced facial hair, triggered feelings of dysphoria for some TGGE individuals.^55^ The discrepancies between their masculine appearance and pregnant physique were described by some as a “complete dysphoria of the body,” and by others as “frightening,” “distressing,” “extremely difficult to handle,” and “body horror.”^48^, ^52^ Coping mechanisms for these feelings included dissociation and detachment from the physical self. Conversely, other studies report pregnancy has a positive influence on body perception. As one person put it: “[…] I didn't bleed [during pregnancy] which was nice, I didn't have hips, I looked like a big trucker boy with a big belly. […] it actually kind of worked for me […].”^51^ However, dysphoric feelings were not experienced by all, and “no two stories were the same.”^57^ In fact, some TGGE people reported that their pregnancy reduced dysphoria and enabled them to build new connections with their bodies and welcome the physical changes.^56^, ^57^ Embracing the “functionality” of their body as a pregnant person reduced feelings of bodily alienation, for example, when they could chest feed their babies. Others responded to changes by viewing their pregnant bodies as a scientific object.^51^

Most studies reported that respondents felt socially isolated, alienated, and not sufficiently supported by friends, family, or medical practitioners.^48^, ^50^, ^56^, ^58^, ^59^ Compounded by experiences of overt and covert discrimination, respondents mentioned pregnancy as a time in which many intersectional factors could affect their mental and physical well‐being, often inducing and perpetuating experiences of exclusion and isolation.^47^, ^48^, ^51^, ^56^, ^58^ One of these factors was the decision to conceal a pregnancy, which came with advantages but also led many trans people to (further) isolate themselves from their social surroundings.^47^, ^48^, ^56^ People presenting as nonpregnant or who went “stealth” reported similar experiences of isolation, in particular feeling invisible in their pregnant trans identities; some said their sense of loneliness was most felt when others, including MNC practitioners, did not acknowledge them as pregnant.^48^, ^56^

### 4.3 Encounters in the maternal and newborn care system

Studies describe both positive experiences and experiences of disempowerment in healthcare encounters. Such encounters took place at two distinct but interwoven levels of the healthcare system: the individual health practitioner level and the institutional level.

Respondents reported positive experiences when health practitioners were aware of the specific challenges of trans pregnancy and normalized their pregnancy, and used gender‐affirmative language in relation to pronouns, chosen names, parental role, and body parts.^61^ All studies found that gender‐inclusive attitudes and skills improved TGGE people's feelings of safety, trust, and recognition in their care. Moreover, although not everyone experienced being misgendered as something that affected them negatively, many felt that being correctly gendered by care practitioners was crucial to their emotional well‐being during pregnancy.^50^, ^58^, ^59^, ^61^ Some respondents reported feeling relieved or more comfortable when healthcare practitioners discussed alternative modes of delivery that could relieve gender dysphoria.^53^

Many study respondents mentioned a lack of practitioner knowledge, skills, and sensitivity about the pregnancy needs of TGGE people as a source of frustration and distress.^48^, ^51^, ^56^, ^57^, ^59^ One study described a respondent being refused perinatal care because of being transgender.^57^ Some respondents had questions about the short‐ and long‐term impact of testosterone use on their reproductive capacity and pregnancy outcomes that their practitioners were unable to answer.^59^ Some practitioners conflated the concepts of gender and sexuality.^61^ Even when practitioners had the best of intentions, they overemphasized the uniqueness and hardship of pregnancy for trans people, making them feel tokenized and even objectified.^59^ It was common for TGGE individuals to provide health practitioners with information about gender, trans bodies, and trans experiences. These respondents felt like they represented an opportunity for practitioners to educate themselves, which alienated them from their care practitioners.^50^, ^61^ Several respondents pointed out that, when practitioners educated themselves about trans‐specific healthcare needs, including pregnancy‐related needs, it relieved them of the burden of educating them at their own expense.^59^

Often, individual practitioners' lack knowledge, gendered bias, or trans‐negative attitudes manifested as micro‐aggressions. Many shared experiences with practitioners who consistently misgendered them or failed to use their chosen name.^56^, ^59^ Some studies describe how respondents were subjected to practitioners' inappropriate comments about their genitals, encountered practitioners who made assumptions about their relationship with their own body, or endured examinations that felt inappropriate or disrespectful.^51^, ^59^ Respondents report being “met by astonishment and prying questions irrelevant to the care.”^51^, ^59^ Pelvic examinations, which respondents often experienced as stressful and therefore preferred to avoid, were not always adequately introduced and their necessity not always properly explained. The lack of opportunity to give full consent made some respondents feel powerless.^61^

In some cases, TGGE people were faced with overt transphobia: refusal of healthcare practitioners to use correct pronouns, questioning of gender identities, or explicit endorsement of binary gender norms and transphobic comments.^48^, ^59^, ^60^, ^61^ Moreover, many had met practitioners of prenatal care who were uncomfortable or unwilling to counsel and treat people other than cisgender women.^48^, ^56^, ^57^ Trans‐negative remarks and attitudes from birth center or hospital personnel often remained uncorrected or unchallenged,^51^, ^59^, ^60^ which caused respondents to feel unsafe in healthcare environments, affecting their care‐seeking; many respondents actively sought out practitioners who were known to have the appropriate skills for TGGE‐inclusive care, for instance, by reaching out to community networks.^50^, ^52^, ^59^ There was little discussion in the included articles of intersections with other aspects of identity that relate to the availability of such community networks, such as geographical aspects, access to digital sources, and cultural factors.

Finally, respondents reported feeling invisible and excluded throughout their pregnancy. They linked their experiences to the absence of TGGE‐inclusive organizational and administrative practices in facilities that provide pregnancy care and counseling. The information and educational material issued by facilities were addressed exclusively to cisgender women.^59^, ^60^ Furthermore, respondents reported a sense of administrative exclusion because of health information systems and processes built on binary gender logic. For instance, records and letters respondents received from facilities often contained their legal name and wrong pronouns.^59^ Such experiences—commencing as purportedly banal and innocuous daily administration—effectively confronted respondents with the fact that, institutionally, their physical and social realities as pregnant people did not exist. Feelings of invisibility and the lack of representation of TGGE pregnancies were linked to distress and worsening feelings of loneliness, and were reported as a factor that affected respondents' well‐being.^48^, ^56^

## 5 DISCUSSION

We conducted a systematic scoping review of the pregnancy experiences of TGGE individuals. Results show that the pregnancy experiences of TGGE people are complex and heterogeneous. Studies reported large interpersonal variations, as well as similarities in perspectives and needs among TGGE people. These findings echo the results of previous papers, reviews, and meta‐synthesis on this topic.^25^, ^27^, ^30^, ^33^, ^62^ However, few authors critically reflected on how pregnancy experiences of TGGE people might intersect with other aspects of identity, such as ethnicity, age, and class, or used critical theory to disentangle negative experiences within the healthcare system. For instance, few studies explicitly understood the experiences of pregnant TGGE people as product of erasure through medical cisnormativity and institutionalized trans‐negativity in clinical care.^1^, ^2^, ^63^ Analysis from a CM perspective puts forward that to optimize reproductive care outcomes of TGGE people, it is necessary to expose and counter anti‐trans and structural cisnormative practices in maternal and newborn care. As evidenced in this review, when unchallenged such ideologies and practices cultivate a care environment that is unsafe and unjust, it further marginalizes TGGE people who are pregnant.

### 5.1 What is a pregnant person?: Exposing the binary gendered foundation of maternal and newborn care

Although positive experiences were reported across all studies and respondents' narratives showed resilience and courage, many accounts reflected persistent and deeply rooted reproductive injustices and exposed the maternal and newborn care environment where these studies were conducted as a location where cultural cisnormative and anti‐trans sentiments might be reproduced and exacerbated.

Dominant cisnormative ideologies and practices, such as those employed and passed down within biomedical healthcare settings and MNC contexts, understand pregnant bodies as essentially cisgender females and pregnancy as an exclusively cisgender female experience, intrinsically but erroneously linking genotypic information to phenotypical realities and socially constructed categories.^21^, ^22^ Such thinking actively unimagines and erases the reality of pregnant TGGE bodies: it renders their pregnancy experiences invisible and even impossible, preventing their experiences from being recognized in consultation rooms, or their narratives and needs addressed as legitimate clinical knowledge in health profession education classrooms.^3^, ^64^, ^65^ As a result, institutional cisnormativity not only produces exclusion and alienation for pregnant TGGE people in relationships with MNC practitioners or facilities but it also embeds bias in administrative and logistic systems. Our findings show how this might lead to administrative difficulties in MNCs, such as not being able to register as a pregnant man.

The current medical discourse surrounding pregnancy is an assembly of linguistics, logistics, and objects which are all designed on the basis of a cisnormative idea of what a pregnant person is*—*and what a transgender person is. By being pregnant, TGGE people implicitly and explicitly challenge both the notion of pregnancy as a cisgender female experience and the notion of TGGE identities as incompatible with pregnancy (i.e., TGGE people as inherently infertile).^66^ Their experiences interrogate fundamentally held “truths” in biomedicine. Exposing these truths as in need of revision might not be without risk, as this review outlines. As the MNC system tries to uphold its cisnormative structure through processes of ignoring, silencing, denouncing, and delegitimizing TGGE pregnant peoples' realities, challenging it—and the practitioners who work inside it—may result in harmful and even violent behavior toward pregnant TGGE people seeking care.^8^, ^22^, ^67^, ^68^ Moreover, medical erasure of trans bodies as potentially pregnant bodies might lead to a cultural definition of TGGE pregnancies as a problem—even a deficit—of the TGGE individual; as Greenfield and Darwin (2020) have it, conceptual invisibility makes “the individual feel not man enough, not trans enough, not pregnant enough, and not safe enough.”^68^

It is important to recognize that erasure in the medical system takes place within a context of, and is compounded by, everyday cultural erasure of TGGE realities, showing up in, for instance, the unavailability of nonfeminized clothing for pregnant persons (“maternity wear”). Moreover, cisnormative and anti‐trans ideologies exist in healthcare contexts in which TGGE people with multiple, intersecting marginalized identities may experience the accumulated impacts of racism, ageism, and classism.^69^, ^70^ The influence of negative experiences within the MNC system therefore cannot be seen separate from the historical context of cissexist and anti‐trans discrimination, as well as individual experiences with transphobia and sexism, and social injustices in a more broad sense.

### 5.2 “Fixing the knowledge”: Reconsidering the relationship between “sex,” “gender,” and “pregnancy,” in midwifery

To address transphobia in midwifery care, several authors have argued that more research into the experiences of TGGE pregnant people is urgent and that midwives' lack of knowledge should be supplemented through practitioner‐level or organization‐level training.^22^, ^25^, ^27^, ^33^ However, our results indicate that more fundamental problems, such as the cisnormative ideological underpinnings of MNC knowledge (whose bodies can, or are allowed to, be pregnant), and subsequent organization of pregnancy care, lie at the heart of exclusion and stigmatization of TGGE individuals in midwifery care and healthcare in general. We argue that more structural, transformative approaches are needed that reconsider the very ontological and epistemological basis of midwifery knowledge about maleness and femaleness.^71^

The need for these transformative approaches is also reflected within broader policy‐related issues and societal debate. Recent political and social developments in both North America and Europe reveal a trend toward the restriction of TGGE rights, and threats to further defund and dismantle TGGE health services and logistics.^72^, ^73^ Writing from the Netherlands, although known for its progressive policies, recent efforts by advocacy organizations to remove institutional barriers to adjust legal gender markers have been obstructed by a coalition of traditionalist political parties, conservative media, and organizations of gender essentialist activists acting on behalf of “concerned citizens.”^74^, ^75^ It takes place in contexts where universal access to reproductive care is being eroded, either through political interventions, lack of funding, or overburdening of the care system, a process which is expected to further exacerbate existing reproductive inequalities.^73^

We acknowledge that more research is needed to better understand the TGGE pregnant people, including those from the “global South,”^1^, ^2^ which requires better data collection on gender identity and sexual orientation.^76^ However, while more inclusive research might add to the body of knowledge about the experiences of TGGE individuals, we believe that “more research” may largely confirm what we already know, and will not trickle down into actual improvements in clinical care.^77^

Moreover, although upskilling midwives and midwifery students may help to meet individual practitioner needs, evidence suggests that the effectiveness of training of this kind is highly dependent on its context.^22^ Research into medical curricula shows that efforts to implement training have been hindered by lack of funding and resources, that they have often been isolated, elective, or extra‐curricular, and that they have depended on local cultural norms about the legitimacy of trans health as an important medical topic. In addition, it has been argued that offering specialized training about TGGE health might further pathologize TGGE experiences by positioning TGGE people as distinct from “regular” patients (i.e., heterosexual and cisgender patients), and therefore as deviant.^64^

As famously argued by Angela Davis, from an abolitionist point of view, it may be said that institutionalized transphobia is inherent to any institution.^78^ One of the key insights of abolition is that it is highly unlikely that institutionalized inequity will stop as a result of reforms or inclusivity.^79^ Merely plugging information about TGGE health outcomes and “best‐care practices” into the existing, fundamentally inequitable, system may therefore, at best, improve individual experiences. In a worst‐case scenario, it may fail to counter trans‐negative ideologies and practices. Moreover, it could confirm midwives' ideas about TGGE reproductive healthcare as a “specialty” occupation instead of a routine skill, further reducing access to and quality of care.^64^ From this perspective, it is therefore essential to focus on the systems underlying reproductive inequity for TGGE people. In other words, we need to reconsider the way in which the relationship among “sex,” “gender,” and “pregnancy” is understood and given meaning to in midwifery: “fixing the knowledge.”^64^, ^80^ Midwives and midwives in training need to acquire knowledge, skills, and reflexivity to investigate the medical and social categories “male” and “female.” We believe an intersectional, or other critical diversity framework, is essential to connect gendered reproductive inequity with sexism, racism, ageism, and classism^81^; therefore, throughout the curriculum and informed by TGGE experts, midwifery educators should invite students to reflect on interconnected systems of reproductive inequity.

Furthermore, we argue that trans discrimination during pregnancy should be understood as obstetric violence.^59^, ^82^, ^83^ Obstetric violence is understood as a system of hierarchical relationships and structures within the obstetric institution, rooted in racial capitalism. Midwifery is not considered part of the obstetric institutions as its practice is rooted in different genealogy, philosophies, values, and foundations; centering on relationality, spirituality, and equity. As such midwifery could even be considered as a counter practice to obstetric violence. However, midwives often depend on and work in the obstetric institution, witnessing and becoming complicit in obstetric violence, as early as in their midwifery training.^84^ Awareness and skills of this kind can help all midwives to not only develop trusting relationships with clients of all genders but also become abolitionist agents of change toward just pregnancy care.^62^, ^82^, ^85^, ^86^ Current implementation challenges involve identifying pedagogical frameworks and/or theoretical paradigms that could guide such efforts. Further research is needed into the potential of transfeminist, abolitionist, or decolonial frameworks in this context, and good practices and examples are already ubiquitous in the field of “women's health.”^62^, ^65^, ^87^

## 6 CONCLUSION

This systematic scoping review provides supportive evidence for pervasive trans discrimination in pregnancy care in “high‐income countries,” which further adds to evidence about structural factors as a cause for inequity in care and care outcomes. Considering the background of attacks on trans rights, we argue that the implementation of CM perspectives in midwifery education could provide a necessary counterweight to anti‐trans ideologies and practices. This requires frameworks for exposing and challenging cisnormative ideas and practices in MNC systems, reconsidering the way in which the relationship among “sex,” “gender,” and “pregnancy” is understood and given meaning to in midwifery, and applying an intersectional lens to investigate the relationship between TGGE identity and reproductive injustice, and how it might affect the realities of TGGE people with multiple, intersecting marginalized identities who may experience the accumulated impacts of racism, ageism, and classism. Future research should identify pedagogical frameworks that are suitable for guiding implementation efforts.

## FUNDING INFORMATION

No funding was received to assist with the preparation of this manuscript.

## CONFLICT OF INTEREST STATEMENT

N/A.

## Supporting information


Data S1.

Data S2.

## Data Availability

Data sharing not applicable to this article as no datasets were generated or analysed during the current study.

## Footnotes

* We use the term TGGE individuals to refer to individuals whose gender identity does not align with the gender assigned to them at birth, including non‐binary, gender fluid, agender and genderqueer individuals. These identities intersect and overlap and might be given meaning differently by different individuals and even within individuals at different times. We therefore chose not to define these identities, but instead discuss the implications of different gender expressions and treatment choices on clinical care in the context of midwifery care.

† Going stealth in the context of TGGE individuals refers to a person who has physically transitioned to not disclose their transition history; instead presenting as a cisgender person, in this case, cisgender man.
