# Supplementary material for: Pregnancy experiences of transgender and gender‐expansive individuals: A systematic scoping review from a critical midwifery perspective
Source: Birth. 2024 May 20;52(3):421–34. doi: 10.1111/birt.12834 (PMC12434226; doi:10.1111/birt.12834)
Supplement: Supplementary file 1 — Data S1. [file BIRT-52-421-s001.docx]

# **S1. Search strategies**

# **PubMed History and Search Details May 31, 2023**

| **Search** | **Query** | **Results** |
| --- | --- | --- |
| #4 | **#1 AND #2 AND #3** | 1,546 |
| #3 | **"Patient Satisfaction"[Mesh] OR "Attitude"[Mesh:NoExp] OR satisf*[tiab] OR prefer*[tiab] OR perspective*[tiab] OR perception*[tiab] OR perceiv*[tiab] OR experienc*[tiab] OR opinion*[tiab] OR narrative*[tiab] OR wish*[tiab] OR desire*[tiab] OR need*[tiab] OR attitude*[tiab] OR stories[tiab] OR story[tiab] OR account*[tiab] OR challeng*[tiab] OR problem*[tiab] OR barrier*[tiab] OR concern*[tiab] OR belief*[tiab] OR consider*[tiab]** | 9,331,268 |
| #2 | **"Prenatal Care"[Mesh] OR "Perinatal Care"[Mesh] OR "Pregnancy"[Mesh] OR "Delivery, Obstetric"[Mesh] OR "Midwifery"[Mesh] OR "Nurse Midwives"[Mesh] OR "Obstetrics"[Mesh] OR prenatal care[tiab] OR perinatal care[tiab] OR pregnan*[tiab] OR labor[tiab] OR labour[tiab] OR birth*[tiab] OR childbirth*[tiab] OR midwife*[tiab] OR midwiv*[tiab] OR birth attendant*[tiab] OR obstetric*[tiab] OR gynaecolog*[tiab] OR gynecolog*[tiab] OR parturition*[tiab]** | 1,539,528 |
| #1 | **"Transgender Persons"[Mesh] OR "Transsexualism"[Mesh] OR transman[tiab] OR transmen[tiab] OR transsex*[tiab] OR trans-sex*[tiab] OR transgender*[tiab] OR gender identit*[tiab] OR gender nonconform*[tiab] OR gender identity disorder*[tiab] OR gender dysphor*[tiab] OR gender incongruent*[tiab] OR gender varian*[tiab] OR gender ambiguit*[tiab] OR gender minorit*[tiab] OR two-spirit*[tiab] OR non-binary[tiab] OR nonbinary[tiab] OR genderqueer*[tiab] OR gender divers*[tiab] OR gender-affirming[tiab] OR transmasculin*[tiab]** | 21,407 |

# **Embase.com History and Search Details May 31, 2023**

| **Search** | **Query** | **Results** |
| --- | --- | --- |
| #6 | **#4 NOT ('conference abstract'/it OR 'conference review'/it)** | 1,665 |
| #5 | **#4 AND ('conference abstract'/it OR 'conference review'/it)** | 804 |
| #4 | **#1 AND #2 AND #3** | 2,469 |
| #3 | 'patient satisfaction'/exp OR 'attitude'/exp OR 'perspective'/exp OR 'desire'/exp OR 'barriers'/exp OR 'experience'/exp OR satisf*:ti,ab,kw OR prefer*:ti,ab,kw OR perspective*:ti,ab,kw OR perception*:ti,ab,kw OR perceiv*:ti,ab,kw OR experienc*:ti,ab,kw OR opinion*:ti,ab,kw OR narrative*:ti,ab,kw OR wish*:ti,ab,kw OR desire*:ti,ab,kw OR need*:ti,ab,kw OR attitude*:ti,ab,kw OR stories:ti,ab,kw OR story:ti,ab,kw OR account*:ti,ab,kw OR challeng*:ti,ab,kw OR problem*:ti,ab,kw OR barrier*:ti,ab,kw OR concern*:ti,ab,kw OR belief*:ti,ab,kw OR consider*:ti,ab,kw | 12,414,131 |
| #2 | 'prenatal care'/exp OR 'perinatal care'/exp OR 'pregnancy'/exp OR 'obstetric delivery'/exp OR 'midwife'/exp OR 'obstetrics'/exp OR ‘prenatal care’:ti,ab,kw OR ‘perinatal care’:ti,ab,kw OR pregnan*:ti,ab,kw OR labor:ti,ab,kw OR labour:ti,ab,kw OR birth*:ti,ab,kw OR childbirth*:ti,ab,kw OR midwife*:ti,ab,kw OR midwiv*:ti,ab,kw OR ‘birth attendant*’:ti,ab,kw OR obstetric*:ti,ab,kw OR gynaecolog*:ti,ab,kw OR gynecolog*:ti,ab,kw OR parturition*:ti,ab,kw | 1,884,271 |
| #1 | 'transgender'/exp OR 'transsexualism'/exp OR 'transgenderism'/exp OR 'transsexuality'/exp OR transman:ti,ab,kw OR transmen:ti,ab,kw OR transsex*:ti,ab,kw OR trans-sex*:ti,ab,kw OR transgender*:ti,ab,kw OR ‘gender Identit*’:ti,ab,kw OR ‘gender nonconform*’:ti,ab,kw OR ‘gender identity disorder*’:ti,ab,kw OR ‘gender dysphor*’:ti,ab,kw OR ‘gender incongruent*’:ti,ab,kw OR ‘gender varian*’:ti,ab,kw OR ‘gender ambiguit*’:ti,ab,kw OR ‘gender minorit*’:ti,ab,kw OR two-spirit*:ti,ab,kw OR non-binary:ti,ab,kw OR nonbinary:ti,ab,kw OR genderqueer*:ti,ab,kw OR ‘gender divers*’:ti,ab,kw OR gender-affirming:ti,ab,kw OR transmasculin*:ti,ab,kw | 28,559 |

# **Cinahl (Ebsco) History and Search Details May 31, 2023**

| **Search** | **Query** | **Results** |
| --- | --- | --- |
| S4 | **S1 AND S2 AND 3** | 906 |
| S3 | MH ("Patient Satisfaction+" OR "Attitude+") OR TI (satisf* OR prefer* OR perspective* OR perception* OR perceiv* OR experienc* OR opinion* OR narrative* OR wish* OR desire* OR need* OR attitude* OR stories OR story OR account* OR challeng* OR problem* OR barrier* OR concern* OR belief* OR consider*) OR AB (satisf* OR prefer* OR perspective* OR perception* OR perceiv* OR experienc* OR opinion* OR narrative* OR wish* OR desire* OR need* OR attitude* OR stories OR story OR account* OR challeng* OR problem* OR barrier* OR concern* OR belief* OR consider*) | 2,619,679 |
| S2 | MH ("Prenatal Care" OR "Gender Specific Care" OR "Perinatal Care" OR "Pregnancy+" OR "Delivery, Obstetric" OR "Midwives" OR "Obstetrics") OR TI (“prenatal care” OR “perinatal care” OR pregnan* OR labor OR labour OR birth* OR childbirth* OR midwife* OR midwiv* OR “birth attendant*” OR obstetric* OR gynaecolog* OR gynecolog* OR parturition*) OR AB (“prenatal care” OR “perinatal care” OR pregnan* OR labor OR labour OR birth* OR childbirth* OR midwife* OR midwiv* OR “birth attendant*” OR obstetric* OR gynaecolog* OR gynecolog* OR parturition*) | 431,818 |
| S1 | MH ("Transgender Persons+" OR "Transsexualism" OR "Transsexuals") OR TI (transman OR transmen OR transsex* OR trans-sex* OR transgender* OR “gender Identit*” OR “gender nonconform*” OR “gender identity disorder*” OR “gender dysphor*” OR “gender incongruent*” OR “gender varian*” OR “gender ambiguit*” OR “gender minorit*” OR two-spirit* OR non-binary OR nonbinary OR genderqueer* OR “gender divers*” OR gender-affirming OR transmasculin*) OR AB (transman OR transmen OR transsex* OR trans-sex* OR transgender* OR “gender Identit*” OR “gender nonconform*” OR “gender identity disorder*” OR “gender dysphor*” OR “gender incongruent*” OR “gender varian*” OR “gender ambiguit*” OR “gender minorit*” OR two-spirit* OR non-binary OR nonbinary OR genderqueer* OR “gender divers*” OR gender-affirming OR transmasculin*) | 13,057 |

# **Scopus History and Search Details May 31, 2023**

| **Search** | **Query** | **Results** |
| --- | --- | --- |
| #4 | **#1 AND #2 AND #3** | 4,522 |
| #3 | TITLE-ABS-KEY (satisf* OR prefer* OR perspective* OR perception* OR perceiv* OR experienc* OR opinion* OR narrative* OR wish* OR desire* OR need* OR attitude* OR stories OR story OR account* OR challeng* OR problem* OR barrier* OR concern* OR belief* OR consider*) | 29,379,191 |
| #2 | TITLE-ABS-KEY (“prenatal care” OR “perinatal care” OR pregnan* OR labor OR labour OR birth* OR childbirth* OR midwife* OR midwiv* OR “birth attendant*” OR obstetric* OR gynaecolog* OR gynecolog* OR parturition*) | 2,300,053 |
| #1 | TITLE-ABS-KEY (transman OR transmen OR transsex* OR trans-sex* OR transgender* OR “gender Identit*” OR “gender nonconform*” OR “gender identity disorder*” OR “gender dysphor*” OR “gender incongruent*” OR “gender varian*” OR “gender ambiguit*” OR “gender minorit*” OR two-spirit* OR non-binary OR nonbinary OR genderqueer* OR “gender divers*” OR gender-affirming OR transmasculin*) | 77,882 |
